# Preclinical common data elements for general pharmacological studies (pharmacokinetic sample collection, tolerability, and drug administration). A report of the TASK3‐WG1A General Pharmacology Working Group of the ILAE/AES Joint Translational Task Force

**DOI:** 10.1002/epi4.12721

**Published:** 2023-03-21

**Authors:** Lisa Coles, Patrick A. Forcelli, Karine Leclercq, Anna‐Maria Katsarou, Brian D. Klein, Heidrun Potschka, Rudiger Koehling, Lauren Harte‐Hargrove, Aristea S. Galanopoulou, Cameron S. Metcalf

**Affiliations:** ^1^ Department of Experimental and Clinical Pharmacology The University of Minnesota, College of Pharmacy Minneapolis Minnesota USA; ^2^ Department of Pharmacology & Physiology Georgetown University Washington District of Columbia USA; ^3^ Department of Neuroscience Georgetown University Washington District of Columbia USA; ^4^ Early Solutions Department UCB Pharma Braine‐l'Alleud Belgium; ^5^ Laboratory of Developmental Epilepsy, Saul R. Korey Department of Neurology Albert Einstein College of Medicine Bronx New York USA; ^6^ National Institute of Neurological Disorders and Stroke, National Institutes of Health Bethesda Maryland USA; ^7^ Institute of Pharmacology, Toxicology, and Pharmacy Ludwig‐Maximilians‐University Munich Germany; ^8^ Oscar‐Langendorff‐Institute of Physiology Rostock University Medical Center Rostock Germany; ^9^ CURE Epilepsy Chicago Illinois USA; ^10^ Isabelle Rapin Division of Child Neurology, Saul R. Korey Department of Neurology Albert Einstein College of Medicine Bronx New York USA; ^11^ Dominick P Purpura Department of Neuroscience Albert Einstein College of Medicine Bronx New York USA; ^12^ Department of Pharmacology and Toxicology The University of Utah, College of Pharmacy Salt Lake City Utah USA

**Keywords:** animal model, case report form, common data element, pharmacology, preclinical

## Abstract

Growing concerns over rigor and reproducibility of preclinical studies, including consistency across laboratories and translation to clinical populations, have triggered efforts to harmonize methodologies. This includes the first set of preclinical common data elements (CDEs) for epilepsy research studies, as well as Case Report Forms (CRFs) for widespread use in epilepsy research. The General Pharmacology Working Group of the ILAE/AES Task Force (TASK3‐WG1A) has continued in this effort by adapting and refining CDEs/CRFs to address specific study design areas as they relate to preclinical drug screening: general pharmacology, pharmacokinetics (PK) and pharmacodynamics (PD), and tolerability. This work has expanded general pharmacology studies to include dose records, PK/PD, tolerability, and elements of rigor and reproducibility. Tolerability testing CRFs included rotarod and Irwin/Functional Observation Battery (FOB) assays. The material provided in the form of CRFs can be delivered for widespread use within the epilepsy research community.

**Key points**

- Common data elements (CDEs) have been incorporated into CRFs to address specific elements of preclinical drug screening studies: general pharmacology, pharmacokinetic/pharmacodynamic, and tolerability studies.
- The CDEs are delineated for high, moderate, and low priorities.
- Tolerability studies can include a variety of assays, and example CDEs/CRFs have been provided for rotarod and FOB (Irwin) assays.
- CDEs and CRFs have been optimized to allow for broad use.

## 1 INTRODUCTION

The International League Against Epilepsy (ILAE) / American Epilepsy Society (AES) Joint Translational Task Force TASK3 working groups (WGs) created the first set of preclinical common data elements (CDEs) for epilepsy research studies to facilitate the use of common terms, harmonize methodologies, and improve data reporting.^1^, ^2^, ^3^, ^4^, ^5^, ^6^ The Pharmacology Working Group of the ILAE/AES Joint Translational Task Force (TASK3‐WG1A) previously defined key CDEs and provided template Case Report Forms (CRFs) for the screening of and research examining pharmacological treatments.^6^ These CDEs were designed for studies in acute seizure models using naïve animals and/or chronic epilepsy models. The impetus behind these efforts was driven by three main factors. First, the growing concerns over reproducibility and translatability of preclinical research to clinical trials and patient populations^7^, ^8^, ^9^; second, to facilitate collaborations across laboratories; and third, to enable comparisons of data obtained in different laboratories.^8^, ^9^, ^10^ Numerous other factors may contribute to translatability such as choice of animal model, species tested, and how each model relates to specific clinical populations. Furthermore, the level of scientific rigor employed (e.g., statistical considerations, study design, and transparency) also affects the relevance of preclinical studies. Although the efforts of the ILAE/AES Joint Translational Task Force have provided valuable tools to promote rigor, reproducibility, and transparency within the epilepsy research community, the need for high quality, reproducible, and translational research using pharmacological approaches in animal models of epilepsy remains.

**FIGURE 1:**
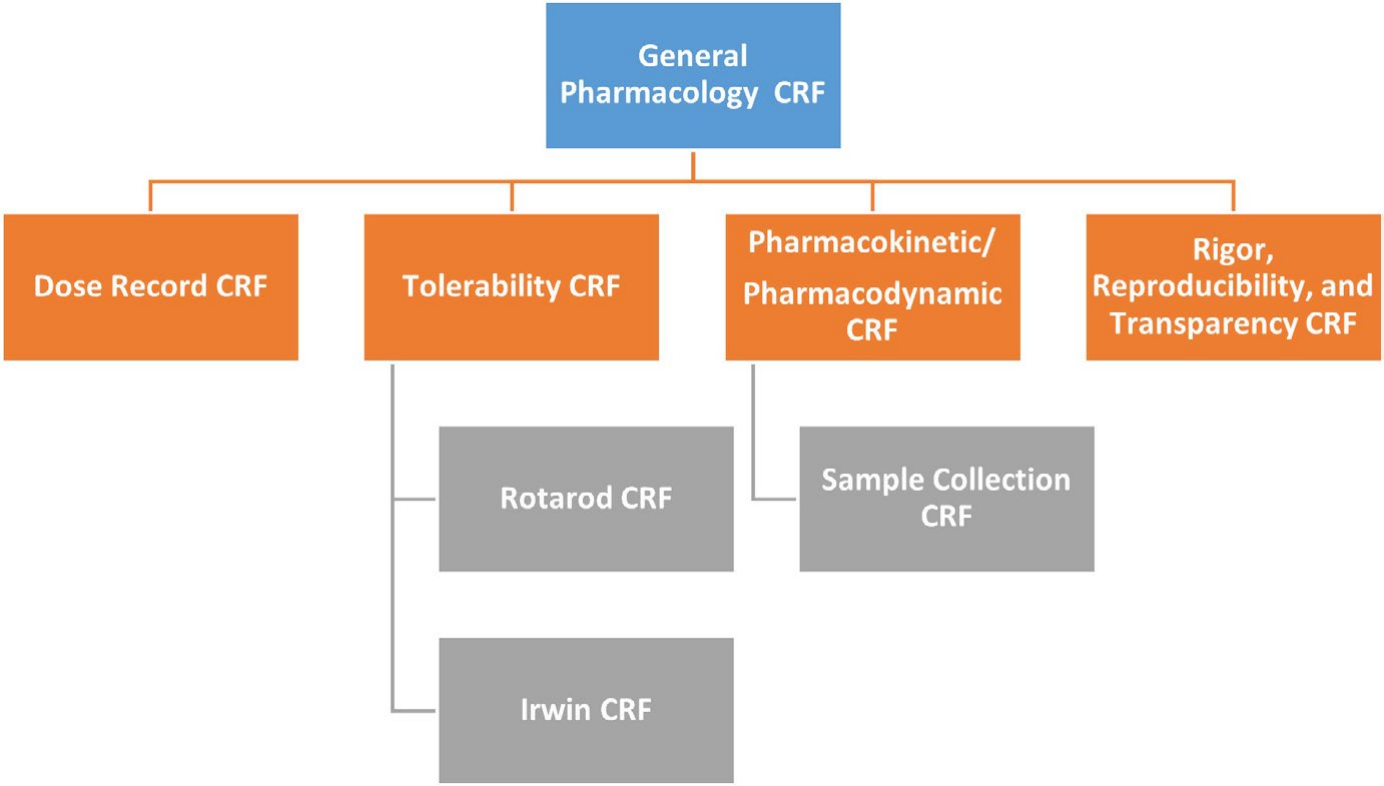
Overview of the general pharmacology working group (TASK3‐WG1A) preclinical case report forms (CRFs).

The General Pharmacology Working Group has continued in this effort by adapting and refining CDEs/CRFs^6^ to address specific study design areas as they relate to preclinical drug screening: general pharmacology, pharmacokinetics (PK) and pharmacodynamics (PD), and tolerability. Definitions of key terminology are provided in Table S1. This companion paper complements the work authored by Barker‐Haliski et al.^6^ and outlines updated or newly developed preclinical CRFs/CDEs pertaining to general pharmacology, dosing records, PK/PD, and tolerability studies (Figure 1). The material presented herein is directed toward laboratories conducting preclinical pharmacology experiments on therapies for epilepsy and seizures. Furthermore, it is important to recognize that individual academic or pharmaceutical laboratories may have already existing standardized reporting strategies related to the CDEs presented herein. Ideally, the material and approaches of this manuscript will aid in harmonization between academic, pharmaceutical, and other entities.

### 1.1 General pharmacology

The general pharmacology CDEs/CRFs were adapted and expanded to support a variety of preclinical pharmacology studies including PK/PD, efficacy, and tolerability profiling. In the general core pharmacology CRF (Module 1), CDEs were selected to provide critical information surrounding drug or active pharmaceutical ingredient (API) formulation and preparation, dosing parameters, dose–response parameters (e.g., median effective and tolerable doses, ED50 and TD50, respectively), randomization, and study design. Of note, tolerability herein refers generally to behavioral impairment, though a variety of parameters may be used to determine median tolerable or median toxic doses (TD50s). A separate CRF was generated to capture dosing history (Module 2; General Core Pharmacology Dosing Records). This includes information for single or repeat doses, as well as various routes of administration (intraperitoneal (IP), per os (PO), intravenous (IV), subcutaneous (SC), intramuscular (IM), intranasal (IN), and intracerebroventricular [ICV]), dose units, and time(s) of administration.

### 1.2 Pharmacokinetic/Pharmacodynamic (PK/PD)

The incorporation of sample collection for bioanalysis and identification of critical PK parameters (defined in Table S1) such as half‐life, maximal concentration, area‐under‐the‐curve (AUC) paired with efficacy, or other behavioral measures (i.e., pharmacodynamics endpoints) is an important component of preclinical pharmacology studies. These outcomes may be impacted by sample handling and processing techniques. CRF Module 3: Pharmacokinetics (Blood and Tissue Sampling) was designed to obtain critical information in the conduct of such studies including study design and blood and tissue sampling. The TASK3‐WG1A identified key parameters to capture dosing and sample collection parameters including biological matrix type (plasma, brain, etc.), history, processing, and storage.

### 1.3 Tolerability

While there are several ways of assessing tolerability in preclinical research,^11^, ^12^, ^13^ our efforts centered around two commonly used tests: the rotarod assay^12^, ^14^, ^15^, ^16^ (CRF Module 4: Rotarod) and the Functional Observation Battery (FOB), also commonly referred to as the Irwin test^17^, ^18^, ^19^ (CRF Module 5: Functional Observation Battery [Irwin Test]). The Irwin test was originally developed in mice,^19^ whereas the FOB is used for a variety of species, most notably rats.^17^, ^18^ The Irwin assay is a tolerability test applied in CNS drug development and is used to obtain comprehensive general information about the impact of the investigational therapy on various aspects of nervous system function (central, peripheral, and autonomic). Phenotypic characterization from the Irwin test can be used to select and apply domain‐specific and sensitive assays to obtain more detailed information when an untoward drug effect is observed. The assay includes a variety of behavioral endpoints and can be used not only for drug studies but also for assessing phenotypic variations in transgenic animals.^20^

The rotarod assay is used for both mice and rats and can incorporate several variations in testing parameters including rod rotation speed, acceleration, and time of testing (i.e., time after drug administration). Therefore, the CDEs/CRFs developed by the TASK3‐WG1A are designed to incorporate the various testing options that may occur in the FOB and rotarod assays. While there are additional behavioral assays to assess tolerability, the rotarod and FOB are included due to their common use in a variety of settings and will serve as an example of how CDEs and CRFs can be implemented for other behavioral tolerability tests when necessary.

In the sections below, we describe and discuss the relevant CDEs required for general pharmacology studies in animal models of epilepsy, including PK/PD and tolerability studies.

## 2 APPROACH

The TASK3‐WG1A members met frequently under the direction of the Task Force to develop CDEs around the critical areas outlined above. Furthermore, subgroups were assigned around general pharmacology, PK/PD, and tolerability to prepare CRFs. Each subgroup was tasked with defining and describing CDEs and drafting CRFs, which were reviewed within the WG at large. The WG discussions helped to refine and optimize CRFs, which were then shared with the Task Force as a whole for additional comments. Our process was first to define and describe CDEs and next to determine by consensus whether a given CDE was deemed to be high priority (highly recommended) or lower priority in terms of both data collection and subsequent reporting. It is recognized that these priority levels may be modified by the researchers and may also depend on stage and type of study and laboratory resources. All CDEs were discussed by the multidisciplinary team, and final decisions were made by consensus. A quorum of at least five team members were present at discussions. Members not present were asked to respond by email. In making these determinations, we considered whether a given CDE (1) would improve the quality of the study, (2) was feasible to implement within preclinical settings, and (3) was redundant with the CDEs and CRFs defined by other WGs. One of our principal goals in this endeavor was to improve and encourage implementation and usability of these CRFs.

## 3 GENERAL PHARMACOLOGY

We suggest that CDEs/CRFs are most useful when they are both thorough and efficient. As a result, we have produced and updated CRFs that include a full range of data elements for general pharmacology, PK and PD studies, and tolerability. In this section, we highlight our consensus on the most important features of each CRF, to emphasize the key components necessary for rigorous record keeping and transparency in reporting. While the overall objective of the CDE initiative is to ensure the quality of preclinical research, we recognize that potential recording and reporting burden needs to be balanced with the needs and complexities of a particular study. For example, while multisite preclinical studies might use a more expansive CRF, a study conducted in an individual laboratory may use a more restricted CRF. Thus, while implementation will almost certainly vary across sites, some features are essential components of rigorous reporting. These CDEs are outlined for the Core Pharmacology CRF in Table 1. They focus on ensuring high quality reporting to reproduce dosing formulations, the key features of the study design, and key features of investigational agent administration. For dosing records, we propose that all CDEs are high priority and recommend they be recorded in all cases.

**TABLE 1:** High‐priority common data elements—core pharmacology treatment administration.

Core pharmacology (test substance)	Core pharmacology (study design)	Core pharmacology (drug administration)
Test substance	Study type	Dosing paradigm
Origin/Manufacturer	Experimental/comparator groups	Route of administration
Lot	Seizure history	Doses and dose volumes
Salt/Free base	Blinding	Time of administration
Formulation	Randomization	
Concentration		
Vehicle/excipients		
Formulation pH		
Purity		

A component of our revisions to the previous core pharmacology CDEs and associated CRF was to incorporate the additional features needed, while minimizing redundancy. While many of the proposed CDEs can be common across several assay types, parameters outlined herein describe specific test elements and can be linked to other core CDEs/CRFs. We recognize that, particularly in the context of therapy screening studies, record‐keeping should not be more burdensome than the associated experiments. To reduce the burden of record‐keeping, we have separated out “study level” CDEs, which can be completed once per study and be repeated across all or a subset of study subjects as applicable, and “subject level” CDEs, which are completed on a per subject basis. We intentionally created the general pharmacology CRF (Module 1) as “study level” while the remaining CRFs were created as “subject level.” The subject level forms could be used for each individual animal. If repeated specimen collections are done from an animal at different time points, or different specimens are collected from the same animal, the CRF form can include entries for different time points or different specimens. If the CRF is in printed form, the PK CRF can be adapted to include all this information. If a CRF is used in electronic format (e.g., spreadsheet or other data management system), it is possible to utilize multiple entries for the different specimens or timepoints, so that the specific information for each collected specimen is entered.

Therefore, the goal of the revised material presented herein was to streamline the use of CRFs as much as possible and aid in implementation and wider use across laboratories worldwide.

## 4 PK/PD

### 4.1 Case Report Form: Pharmacokinetics (Blood and tissue sampling) (Module 3; Appendix S1)

This CRF covers three main areas of PK studies: sample collection, sample processing, and sample storage. This form is intended for each individual animal; however, spreadsheets could be used to capture these same CDEs. We outline the high‐priority data elements for the PK CRF in Table 2. These were selected to ensure key features of sample collection and storage were recorded.

**TABLE 2:** High‐priority common data elements—pharmacokinetic parameters.

General settings	Collection records	Core pharmacology (drug administration)
If matrix—tissue, blood, or cerebrospinal fluid (CSF)	Date/Time of dose(s)	Processing method
If blood—serum/plasma/red blood cells / whole blood	Nominal collection date(s)/time(s)	Storage conditions
Container additives	Actual collection date(s)/time(s)	

Established guidelines and procedures should be used for tissue collection^21^, ^22^, ^23^ and, as described below, there are several important considerations when collecting biological tissue. This CRF includes specific information for data collection in the *General Settings for Blood and/or Tissue Sampling* subsection. An essential element that needs to be specified for PK studies is the biological matrix. The matrix will most often refer to brain tissue, blood/plasma, or cerebrospinal fluid (CSF) in these studies. Brain tissue can be either the whole brain or parts of it. Given that drugs are often distributed differently between whole blood, serum, plasma, and red blood cells, recording the type of sample collected and subsequently processed is a high‐priority CDE. In addition, we recommend that the location(s) of blood collection are described (e.g., cardiac puncture, tail vein, saphenous or dorsal pedal vein, jugular, trunk, or retroorbital) as well as if an indwelling catheter was used for sampling. Details about anesthesia, if used, should be documented as well. Information about sample collection methods such as container type and preservatives/additives is also important to note as additives may impact the subcomponent of the matrix that can be efficiently measured (e.g., heparin or ethylenediaminetetraacetic acid [EDTA]). In cases of organ/tissue sampling, it is also critical to document whether the harvested tissue was perfused (e.g., with saline to purge blood) prior to tissue collection. If so, details referring to the procedure of the perfusion should be included as part of this CRF. Blood volumes and tissue weights to be collected will vary from study to study and from animal to animal. Adequate volumes/weights should be determined by the number of assays to be performed, volume requirements, and processing instructions. For example, in brain tissue, researchers should consider whether perfusion of brain prior to harvesting tissue or dissection is required. This will be determined by the study objectives and assay methods.

Following the *General Settings for Blood and/or Tissue Sampling* is the *Dose and Sample Collection Records* subsection. This form can be repeated for each sample and matrix or the CDEs can be incorporated into a spreadsheet. The date of dose(s), as well as the time(s) of dose(s), is key information for the study. The same holds true for the nominal time of the sample collection, which refers to the planned scheduled time for sample collection that may be different than the actual time the collection takes place during the experiment. All the above‐described data can be used as additional data to the *CRF Module 1: General Core Pharmacology* when dosing and PK sample collection records are needed.

The next two major subsections of this CRF deal with sample processing and sample storage, respectively. An important CDE for sample processing is the method of tissue processing. Among the most notable methods are snap freezing, tissue homogenization for the isolation of compounds of interest, and centrifugation. Centrifugation is the process during which substances of different densities are separated using a centrifuge. If that is the chosen method, centrifugation parameters (e.g., duration, speed, and temperature) should be defined. Snap freezing is the method of rapid cooling of a substance for preservation purposes, for which time of processing should be included. If the sample refers to plasma or serum, the occurrence of hemolysis should be reported. This is particularly important for drugs that sequester in red blood cells. Similarly, if brain tissue is collected, it is important to note whether the sample contains potential blood contaminants. Other important elements include the number and volume of aliquots used, the serum and/or plasma volume, and the tissue weight. The final subsection describes sample storage conditions, such as whether the sample was kept at room temperature, refrigerated, or frozen (−20°C vs −80°C).

## 5 TOLERABILITY (APPENDIX S1: TOLERABILITY)

A wide array of behavioral tests can be used to assess various aspects of drug tolerability and side effect profiles. Many of these tests have already been described in detail in the companion paper to the TASK3 Behavior WG of the ILAE/AES Joint Translational Task Force (see: Mazarati et al.^4^), albeit in the context of phenotyping comorbidities of epilepsy in preclinical models. There are several behavioral tolerability assays that may be employed in preclinical pharmacology studies. These include the Open Field Test, The Footfall Test, and Automated Phenotyping. Importantly, we have not provided an extensive list or set of CRFs for tolerability. Rather, we instead focused on two of the most common—the rotarod and Irwin test (FOB)—to use as examples of how CRFs might be developed for such assays. We outline the high‐priority data elements for the Tolerability CRFs in Table 3.

**TABLE 3:** High‐priority common data elements—tolerability parameters (applicable to rotarod and Irwin CRFs).

General settings (for both rotarod and Irwin tests)	Rotarod test settings	Irwin test settings
General testing conditions	Equipment parameters	Numerous (note^a^)
General behavioral observations		

^a^ There are numerous behavioral elements within this test and critical elements go beyond the scope of this table.

### 5.1 Rotarod (CRF Module 4; Appendix S1: Rotarod)

The rotarod test is widely used as a measure of minimal motor impairment following administration of neuroactive compounds. When coupled with efficacy measurements in preclinical screening models (see Barker‐Haliski et al.^6^), the motor impairment in the rotarod test allows for estimation of the therapeutic index of candidate compounds. The rotarod, originally described as a “rolling roller apparatus,” was developed as a screening tool for motor impairment, when testing novel antiseizure agents,^24^ as well as other drug‐induced movement impairments. The task consists of placing an animal (typically a mouse or rat) on a rotating rod and measuring its latency to fall from the rod. In the more than 60 years of use, rotarod impairment has been demonstrated for a wide range of agents including benzodiazepines, barbiturates, ethanol, neuroleptic agents, sodium channel blockers, and a wide range of other antiseizure medications.^25^, ^26^

While a simple test, a range of variables have profound impact on rotarod performance, including the diameter of the rod,^27^ rod texture, the use of a fixed rate vs. accelerating rod,^28^ habituation procedures, and examination of learning vs. single trial tests.^29^ All of these task parameters can not only impact baseline performance in naïve animals, but also response to drugs.^27^ We identified the critical data elements for accurate record‐keeping and reporting that captured the common variables that differ between studies. Most of these variables are “Study Level” variables, which only need to be completed once per study (e.g., the model of the rod, the diameter, texture, and speed). The critical dependent variable (“Subject Level”) is the latency to fall from the rod.

### 5.2 Functional observation battery or irwin test (CRF Module 5; Appendix S1: Functional Observation Battery [Irwin Test])

A comprehensive battery for the evaluation of behavioral and neurological side effects of drugs was originally described several decades ago^19^ and has since been used extensively as a preclinical therapy development tool by pharmaceutical, academic, and government laboratories. After further refinement of these techniques and standardization,^17^ the FOB is widely accepted as a broadly applicable tool for the assessment of side effects in laboratory rodents.

The FOB includes evaluation and quantification of multiple parameters across several neurological areas including autonomic, neuromuscular, sensorimotor, and other behaviors. Importantly, these behaviors are observed in groups of four or more animals, generally rats, and across a dose range. The CDEs described in the accompanying CRF outline critical and suggested parameters for the Irwin test. For most of the CDEs listed, the presence or absence of noted behaviors can be listed as a binary outcome measure (e.g., Yes/No), whereas other criteria may require additional details. For example, if “hyperactivity” is noted, it may be important to describe the nature of hyperactivity such as “wild running” or “hyper locomotion” or “hyper explorative,” in addition to quantifying the number of animals in each treatment group that display such a behavior.

It may also be advantageous for experimenters to group CDEs within a CRF by category. For example, grouping of autonomic behaviors on the CRF may help the experimenter to identify patterns of responses that indicate sympathomimetic activity for a test compound. Pain scoring may also be included, describing common pain behaviors (e.g., self‐destructive biting, vocalizations) and grimace scoring.^30^, ^31^, ^32^

## 6 DISCUSSION

In summary, the efforts of the TASK3‐WG1A have built upon previous efforts and expanded general pharmacology studies to include dose records, PK/PD, tolerability, and other elements to generally improve rigor and reproducibility in epilepsy research. Moreover, the expansive impact of the latter topic required input of additional Task Force WGs but grew out of the collaboration and goals of this group. The undertaking of this work centered around several major goals. The first was to provide the ILAE/AES Joint Translational Task Force, and the larger epilepsy research community, with a comprehensive set of CRFs within the area of general pharmacology. Second, we sought to identify and delineate major CDEs within this area. Third, we carefully considered implementation and utility of these CRFs in practical laboratory settings. Therefore, a major objective of this group was in reducing redundancy, an important step toward application of 3R rules (replacement, reduction, and refinement) in in vivo pharmacology.^33^ Fourth, we sought to implement means by which sampling over time could be applied to these CRFs. And finally, we were mindful of the eventual joining of CRFs with electronic databases to aid in data upload and management. These last two topics are not trivial in that these types of studies can include repeated administrations of the investigational agent, and multiple matrices collected at multiple times. This longitudinal data collection can quickly become complicated. It is important to have a standard method for measuring time and identifying samples in relation to the investigational therapy. Furthermore, paper CRFs need to be easily converted to an electronic database without redundancy or extensive revisions. Additional work is needed to further refine these CRFs and aid in practical implementation in laboratory settings.

Tolerability may be assessed in a variety of ways in association with rodent seizure and epilepsy models. The CDE/CRF examples shown here demonstrate two examples of commonly used assays to determine whether, for example, drug treatment is associated with untoward effects at therapeutically relevant doses. While the design of such studies overlaps with safety pharmacology assessments, it should be noted that there are important differences. For example, dose ranges may be broad in a safety pharmacology evaluation, whereas they may be limited to only doses expected to block or attenuate seizure activity in other studies. Safety pharmacology also extends beyond the rotarod and Irwin/FOB assays described here. Rather, the goals of the tolerability CDEs we identified and the CRFs presented are twofold: (1) to provide a detailed example of how the rotarod and Irwin/FOB assays may be appropriately used for tolerability profiling in association with models of seizures and epilepsy and (2) to provide an example of how other CRFs may be designed if other tolerability assays and corresponding CDEs can be identified.

We worked to identify CDEs and design CRFs based on collective expertise of WG members, comprising several decades of preclinical drug development experience. However, it is noteworthy that the current design of the CRFs as they appear may be limited and open to additional improvements. Therefore, we emphasize the importance of ongoing efforts to add to the work of previous committees. Case Report Forms should be continually revised to meet the needs of the epilepsy research community, while holding standards of data collection and key parameters for specific experimental criteria consistent over time.

Preclinical pharmacology and tolerability studies in laboratory animals carry specific challenges. In vivo experimentation is time‐consuming, costly, and carries special ethical mandates for transparency, minimization of pain and distress, and minimization of numbers of animals used. Incorporation of CDEs is a step to systematically collect, analyze, and share information, thus increasing rigor and reproducibility of preclinical epilepsy research. The use of standardized reporting of study details and results (i.e., through CDEs) provides a way for investigators to share data more efficiently and harmonize the conduct of multicenter studies, and ensure optimal traceability of data. It is a primary goal that these steps will enable more efficient identification of new therapies for epilepsy and seizures, while simultaneously reducing the number of uninformative studies that are conducted and minimizing the number of animals used, thereby reinforcing the 3R principles.

While the CDEs and CRFs shown are specifically centered around preclinical research in epilepsy, many aspects are generalizable and easily adapted to other disease indications. Tolerability assessment, for example, is not unique to preclinical studies in epilepsy and therefore the approach shown is easily transferrable to other disease indications. While CDEs directed primarily at efficacy (e.g., seizure descriptions) are unique to epilepsy, a majority of other CDEs can be applied to other areas. Finally, the CRFs prepared may also serve as a template wherein similar CRFs may be prepared and applied specifically to other disciplines.

One major limitation to the use of CDEs and CRFs in general pharmacology studies, and indeed other studies described in this issue, and elsewhere, will be the practical implementation. Completion of CRFs for discrete experiments will be a time commitment that may require sufficient community engagement, cooperation, funding, and incentives. While electronic repositories may streamline some elements of this data sharing operation, the process of CRF use may still be a major undertaking. We also recognize that the stage, type of study, and laboratory resources will also be used to determine which CDEs will be collected. The efforts of this and other WGs have been to identify CDEs and design CRFs using the collective expertise of committee members in order to provide an up‐to‐date and well‐designed tool for widespread use. However, to capitalize on this resource, community leaders including healthcare providers, researchers, industry leaders, scientific journals, and patient advocates may need to coalesce around a central goal of promoting the use of the CRFs provided herein.

## CONFLICT OF INTEREST STATEMENT

K Leclercq is full‐time employee of UCB Pharma SA. H Potschka received funding for consulting, talks and research collaborations from Eisai, Zogenix, Elanco, Roche, Lario, Exeed Epidarex, Angelini, MSD, Jazz Pharmaceuticals and Galapagos. AS Galanopoulou is the Editor‐in‐Chief of *Epilepsia Open*, associate editor of *Neurobiology of Disease*, and receives royalties from Elsevier, Medlink, and Morgan and Claypool for publications. CS Metcalf is a paid consultant of Sea Pharmaceuticals, LLC. We confirm that we have read the Journal's position on issues involved in ethical publication and affirm that this report is consistent with those guidelines. This report was written by experts selected by the ILAE and the AES and was approved for publication by the ILAE and AES. Opinions expressed by the authors, however, do not necessarily represent the policy or position of the ILAE or AES. This report does not represent the official view of the National Institute of Neurological Disorders and Stroke (NINDS), the National Institutes of Health (NIH), or any part of the US Federal Government. No official support or endorsement of this article by the NINDS or NIH is intended or should be inferred.

## Supporting information


Appendix S1
