# Supplementary material for: Preclinical common data elements for general pharmacological studies (pharmacokinetic sample collection, tolerability, and drug administration). A report of the TASK3‐WG1A General Pharmacology Working Group of the ILAE/AES Joint Translational Task Force
Source: Epilepsia Open. 2023 Mar 21;10(Suppl 1):S9–S17. doi: 10.1002/epi4.12721 (PMC12375975; doi:10.1002/epi4.12721)
Supplement: Supplementary file 1 — Appendix S1 [file EPI4-10-S9-s001.docx]

Supplementary Table 1. Definition of Key Terminology

| Term | Definition |
| --- | --- |
| Common Data Element | Data elements that are common to multiple data sets across different studies^2, 5^ (see also https://cde.nlm.nih.gov/home) |
| Case Report Form | A printed or electronic document designed to record all of the protocol details that are important to report |
| Pharmacokinetics | Describes the absorption, distribution, metabolism, and excretion of drugs (e.g. biologics and small molecular entities). |
| Pharmacodynamics | Describes the response of the body to the drug |
| Half-life | Period of time required for the concentration or amount of drug in the body to be reduced by one-half |
| Area under the curve (AUC) | Integral of a curve that describes the drug concentration as a function of time |
| Maximum concentration | Maximum drug concentration achieved |
| Efficacy | Measure of the ability of an intervention to produce the desired beneficial effect |
| Potency | Measure of drug activity according to the amount required to produce the desired effect |

**Pharmacological studies**

**Case Report Form**

**CRF module 1: General Core Pharmacology**

****** Please note that there are separate CRFs for dosing records and PK sample collection

Date that this CRF was filled out:

Name of person filling out CRF:

Project name/Identifier:

Animal ID:

| **CDE Name** | **Data Collected** |
| --- | --- |
| ***Test Substance / Vehicle Information*** | |
| Test Substance(s) to be tested H |  |
| Test Substance Origin or Manufacturer M |  |
| API Lot# L |  |
| API Purity if known (%) L |  |
| API (Grade; check all that apply) L | ☐ USP ☐ Analytical ☐ USP Secondary ☐ British Pharmacopoeia Standard ☐ European Pharmacopoeia Standard  ☐ Other ☐ Unknown |
| If other, please specify L |  |
| Salt or Free-base? M | ☐ Salt ☐ Free-base ☐ Unknown |
| If salt, note salt factor M |  |
| If multiple forms, specify details H |  |
| Date manufactured if known (MM/DD/YYYY) L |  |
| Date received if known (MM/DD/YYYY) M |  |
| Formulation used (check all that apply) H | ☐ Suspension ☐ Solution ☐ Other ☐ Unknown |
| If other, please specify H |  |
| Formulation Concentration (amt/vol) H |  |
| pH M |  |
| Vehicle/Excipients (include strength/concentration of each) H |  |
| Formulation procedure (check all that apply) H | ☐ Sonication ☐ Mortar and Pestle ☐ Vortex ☐ Heat ☐ Other ☐ |
| Formulation Temperature M (°C) |  |
| Amount of Time for each procedure: M |  |
| Formulation details (Upload protocol or add description) M |  |
| Formulation details uploaded? M | ☐ Yes ☐ No ☐ Unknown |
| ***Study Design*** | |
| What kind of study? Types include both acute and chronic models. (Check all that apply). H | ☐ Antiseizure ☐ Disease modification ☐ Antiepileptogenesis  ☐ Pharmacokinetic/pharmacodynamic ☐ Tolerability ☐ Other |
| Monotherapy or poly-therapy study?    If poly-therapy, how many drugs?  (Link to CRF module 2: General Core Pharmacology Dosing Records) H | ☐ Monotherapy ☐ Poly-therapy ☐ Other  ____ (numerical) |
| Dose-ranging? H | ☐ Yes ☐ No ☐ Unknown |
| If dose-ranging, ED50 or TD50 quantified? M | ☐ ED50 quantified  ☐ TD50 quantified  ☐ Not applicable  ☐ Unknown |
| Experimental/Comparator groups (check all that apply) M | ☐ Other test/treatment group(s)  ☐ Other control/vehicle group(s)  ☐ Other comparator group(s)  ☐ Other historical group(s)  ☐ Same group, baseline  ☐ Same group, crossover  ☐ None |
| If treatment group1, please specify M |  |
| Control/comparator group(s) 2 please specify M |  |
| How many groups? | _____ (numerical) |
| Specify sample size per group? M  (repeatable for each group) | ☐ Yes ☐ No ☐ Not applicable ☐ Unknown |
| Animals with history of seizure? H  (If yes, please refer to seizure phenotyping and monitoring modules) | ☐ Yes ☐ No ☐ Mixed ☐ Unknown |
|  |  |
| Duration animals acclimated to testing room/space (hours) M |  |
| Experimenter blinded to treatment?  See Reproducibility and Rigor CRF H | ☐ Yes ☐ No ☐ Unknown |
| Random allocation of subjects to groups? See Rigor, Reproducibility, and Transparency CRF H | ☐ Yes ☐ No ☐ Unknown |
| ***Drug Administration Protocol (repeat for each drug if drug administration protocol is different).***  ***Refer to separate CRF for dosing records*** | |
| Compound administration paradigm (select all that apply) H | ☐ Single ☐ Repeated ☐ Continuous |
| If repeated or continuous, duration of treatment in days H |  |
| If repeated administration, frequency of treatment H | Every_____(numerical) ☐ Day  ☐ hr  ☐ min  ☐ Custom |
| If custom, please specify: |  |
| If repeated administration, number of doses |  |
| Date / Time of administration (repeatable for each administration) |  |
| Units for frequency of treatment |  |
| Pretreatment study or post-treatment study? H (select all applicable) | ☐ Pretreatment ☐ Post-treatment  ☐ Not applicable ☐ Unknown |
| Route of administration (check all that apply) H | ☐ IP ☐ PO ☐ IV ☐ SC ☐ IM ☐ IN  ☐ ICV ☐ Implanted mini-pump ☐ Medication in water ☐ Medication in food  ☐ Other |
| If other, please specify route |  |
| Dose(s) tested (amt or amt/wt) H | _____(numerical)  ☐ mg/kg  ☐ mol/kg  ☐ other (specify)______ |
| Dose volume (vol or vol/wt) H | _____(numerical)  ☐ ml  ☐ μl  ☐ other (specify)______ |
| If *per os* (p.o.), specify method M |  |
| Was anesthesia used? H | ☐ Yes ☐ No ☐ Unknown |
| If anesthesia used, please specify anesthesia M | ☐ Isoflurane (specify %)  ☐ Ketamine (specify dose, units, route)  ☐ Pentobarbital (specify dose, units, route)  ☐ Other (specify dose, units, route) |
| If diet/drink water, specify target daily amount H | ☐ mg (drug) / L (water)  ☐ vol (drug, concentration) / L (water)  ☐ other (specify amount, concentration) |
| If medication in food, specify recording parameters for dosage calculations H | ☐ mg (drug) / kg (food)  ☐ other (specify amount, concentration) |
| If medication in food or water, specify daily intake of food or water H | ☐ g (food) intake _____  ☐ L (water) intake _____  ☐ other (specify amount of food or water) |
| ***Collection of Biological Samples: Please fill in the appropriate CRFs*** | |
| Collection of blood? See PK/PD CRF M | ☐ Yes ☐ No ☐ Unknown |
| Collection of other tissues/matrix (check all that apply)? See PK/PD CRF M | ☐ Yes ☐ No ☐ Unknown  ☐ Brain ☐ CSF ☐ Other |
| If other, please specify M |  |
| **Additional remarks** |  |

API: active pharmaceutical ingredient(s); CSF: cerebrospinal fluid; ED50: median effective dose; ICV:intracerebroventricular; IP: intraperitoneal; IM: intramuscular; IN: intranasal; IV: intravenous; PD: pharmacodynamics; PK: pharmacokinetic; PO: oral;; SC: subcutaneous; TD50: median toxic dose; USP: United States Pharmacopeia

**General instructions**

Please check each box where applicable. If none of the predetermined options is appropriate use the default space to specify your answer. The form is to be filled in for one individual animal. This form collects information on the general study design. For information about the performance of the specific test used for the drug study use the specific CRFs to the tests/models of interest (e.g. maximal electroshock, MES). Make sure that the number of CRFs belonging to the same animal and study are indicated and these CRFs can easily be identified.

**Pharmacological studies**

**Case Report Form**

**CRF module 2: General Core Pharmacology Dosing Records**

Date that this CRF was filled out:

Name of person filling out CRF:

Project name/Identifier:

Animal ID:

| **CDE Name** | **Data Collected** |
| --- | --- |
| ***Dosing Records (repeat for each administration)*** | |
| Randomization ID (if applicable) M |  |
| Test Substance or Test Group (i.e. drug(s) if known or blinded group identifier) M |  |
| Animal weight (e.g. grams (g)) H | ☐ g ☐ kg ☐ Other ☐ Not applicable |
| If other weight unit used, please specify H |  |
| Route of administration (check all that apply) H | ☐ IP ☐ PO ☐ IV ☐ SC ☐ IM ☐ IN ☐ ICV ☐ Implanted mini-pump;  ☐ Medication in water  ☐ Medication in food ☐ Other |
| If other, please specify H |  |
| Date of administration H |  |
| Time of administration H |  |
| Timepoint (if applicable) M |  |
| Dose and units (amt/wt, e.g. mg/kg) H | _____(numerical)  ☐ mg/kg  ☐ mol/kg  ☐ other (specify)______ |
|  |  |
| Dose and units (amt, e.g. mg) H |  |
| Dose and units (volume/wt, e.g. mL/kg) H |  |
| Dose and units (volume, e.g. mL) H |  |
| **Additional remarks** |  |

IP: intraperitoneal; ICV: intracerbroventricular; IM: intramuscular; IN: intranasal; IV: intravenous; PO: oral; SC: subcutaneous; amt: amount, wt: weight

**General instructions**

Please check each box where applicable. If none of the predetermined options is appropriate use the default space to specify your answer.

The form is to be filled in for one individual animal.

Make sure that the number of CRFs belonging to the same animal and study are indicated and these CRFs can easily be identified.

**Pharmacology Studies**

**Case Report Form**

**CRF module 3: Pharmacokinetics (Blood and Tissue sampling)**

Date the CRF was filled out:

Name of person filling out CRF:

Study Site:

Project Name/Identifier:

Animal ID:

Randomization ID:

| **CDE** | **Data Collected** |
| --- | --- |
| ***General Settings for Blood/Tissue Sampling*** | |
| Biological Matrix. Select only one. Complete a CRF for each matrix H | ☐ Blood ☐ CSF ☐ Brain tissue ☐ Other* |
| Brain tissue M | Whole brain ☐ Other brain region (specify brain region _________) not applicable |
| If other brain region, please specify M |  |
| If other* type of sampling please specify H |  |
| If blood sampling please specify (check all that apply) H | ☐ Serum ☐ Plasma ☐ Red blood cells ☐ Whole blood  ☐ Other |
| If other type of blood sampling, please specify H |  |
| Blood collection location(s) M | ☐ Heart puncture ☐ Tail vein ☐ Saphenous vein ☐ Dorsal pedal vein ☐ Jugular ☐ Ocular ☐ Catheter* ☐ Trunk  ☐ Other |
| If catheter*, please specify location M |  |
| If other blood collection location, please specify M |  |
| Anesthesia H | ☐ Yes ☐ No ☐ Unknown |
| If anesthesia used, please specify details M |  |
| Sample collection method(s) additional details M | Enter additional details as needed or SOP number |
| Sample collection method(s) SOP number if available M |  |
| Sample collection container(s) used (check all that apply) M | ☐ Vacutainer tube  ☐ Cryovial  ☐ Centrifuge tube  ☐ Other  ☐ Unknown |
| If other sample collection container(s) used, please specify M |  |
| If Vacutainer tube, please specify L |  |
| If Cryovial, please specify L |  |
| If Centrifuge tube, please specify L |  |
|  |  |
| Container additive(s) (check all that apply) H | ☐ Lithium heparin ☐ Sodium heparin; ☐ K2EDTA; ☐ K3EDTA; ☐ SST ☐ PST ☐ Citrate ☐ Other ☐ Unknown |
| If other, please specify H |  |
| Tissue perfusion prior to harvest H | ☐ Yes ☐ No ☐ Unknown |
| If there was tissue perfusion prior to harvest, please specify details M |  |
| ***Dose and Sample Collection Records (Repeat for multiple samples and matrices)*** | |
| Date of Dose (MM/DD/YYYY) H |  |
| Time of Dose H |  |
| Sample ID number (if applicable) M |  |
| Date of Collection (MM/DD/YYYY) H |  |
| Sample Collection Time Point H |  |
| Nominal Time L |  |
| Actual Time H |  |
| Sample Volume or Weight L | _____(numerical)  ☐ mg  ☐ ml  ☐ other (specify)______ |
| Processing method (check all that apply) M | ☐ None ☐ Centrifugation ☐ Tissue homogenization ☐ Snap freeze  ☐ Other ☐ Unknown |
| If other processing method used please specify M |  |
| Centrifugation Speed RPMs: _____; RCF/g:________ M |  |
| Centrifugation Time M |  |
| Centrifugation Temperature M | ☐ Room temperature ☐ Refrigerated  ☐ Other |
| Define room or refrigerated temperature L (°C) |  |
| If other please specify M |  |
| Processing Methods. Enter additional details as needed or SOP number M |  |
| Storage containers used (check all that apply) M | ☐ Cryovial  ☐ Centrifuge tube  ☐ Other  ☐ Unknown |
| If cryovial storage containers used, please specify M |  |
| If centrifuge tube storage containers used, please specify L |  |
| Number of aliquots L |  |
| Volume of aliquots L |  |
| Plasma/Serum Volume L |  |
| Tissue  Vial weight empty M |  |
| Tissue  Vial weight + tissue M |  |
| Tissue weight M | _____(numerical)  ☐ mg  ☐ g  ☐ other (specify)______ |
| Storage Conditions M |  |
| Sample Location M |  |

**General instructions**

Please check each box where applicable. If none of the predetermined options is appropriate use the default space to specify your answer. The form is to be filled in for one matrix. This form collects information on the pharmacokinetic study. Make sure that the number of CRFs belonging to the same animal and study are indicated and these CRFs can easily be identified.

**Tolerability Studies**

**Case Report Form**

**CRF Module 4: Rotarod**

Date that this CRF was filled out:

Name of person filling out CRF:

Project name/Identifier:

Animal ID:

| **CDE** | **Data collected** |
| --- | --- |
|  | |
|  | |
| Model H |  |
| Source H |  |
| Rod diameter in (cm) M |  |
| Rod texture M |  |
| Constant or Accelerating H | ☐ Constant ☐ Accelerating ☐ Unknown |
| Starting speed (RPM) H |  |
| Test duration in (sec) H |  |
| Acceleration rate in (RPM/sec) H |  |
| Ramp description M | ☐ constant change  ☐ step-wise change  ☐ other (specify)______ |
| Total number of trials M |  |
| Starting rotation direction relative to animal M | ☐ Forward ☐ Reverse |
| Animal is placed back on the rod after a fall? M | ☐ Yes ☐ No |
| ***Outcome data*** |  |
| Latency to fall (for each trial) in sec H |  |
| Number of falls H |  |

**Instructions**: Please check boxes where applicable. If none of the predetermined options is appropriate use the default space to specify your answer. This form is to be filled in for one individual animal.

**Tolerability Studies**

**Case Report Form**

**CRF Module 5: Functional Observation Battery (Irwin Test)**

**recommendations for core parameters; additional components can be added and some CDEs can be scored (i.e. rather than yes/no)*

Date that this CRF was filled out:

Name of person filling out CRF:

Project name/Identifier:

Animal ID:

| **CDE** | | **Data collected** |
| --- | --- | --- |
|  | | |
| ***Test settings*** | | |
| Behavioral Descriptions (for adult animals) M |  | |
| Death H | ☐ Yes ☐ No | |
| If death, date (MM/DD/YYYY) of death M | ______________ (date)  ☐ Unknown | |
| If death, time of death M | _______________(time)  ☐ Unknown | |
| General body condition description M |  | |
| Was scoring rubric used? M | ☐ Yes ☐ No | |
| Total score H | _____(arbitrary units)  Other (specify)_____ | |
| If scoring rubric used, attach scoring rubric and/or description M |  | |
| Body tone (abnormal) including hyper- or hypo-tonia H | ☐ Yes* ☐ No ☐ Unknown | |
| If yes*, please describe M |  | |
| Body position (abnormal) Including body flattening M | ☐ Yes* ☐ No | |
| If yes*, please describe L |  | |
| Seizure-like behaviors and/or convulsions H | ☐ Yes* ☐ No  Date/time observed _____ | |
| If yes, date observed (MM/DD/YYYY) | ______________ (date)  ☐ Unknown | |
| If yes, time observed | _______________(time)  ☐ Unknown | |
| If yes*, please describe M  (link to seizure phenotyping (Melissa et al) or monitoring CRFs (Ono et al)) |  | |
| Myoclonic jerks H | ☐ Yes ☐ No | |
| Straub tail H | ☐ Yes ☐ No | |
| Diarrhea H | ☐ Yes* ☐ No ☐ Unknown | |
| If yes*, please describe M |  | |
| Constipation M | ☐ Yes* ☐ No ☐ Unknown | |
| If yes*, please describe L |  | |
| Salivation H | ☐ Yes ☐ No ☐ Unknown | |
| Piloerection H | ☐ Yes ☐ No | |
| Excessive urination H | ☐ Yes ☐ No ☐ Unknown | |
| Urinary staining H | ☐ Yes ☐ No ☐ Unknown | |
| Red tears M | ☐ Yes ☐ No ☐ Unknown | |
| Ataxia H | ☐ Yes ☐ No | |
| Sedation H | ☐ Yes ☐ No | |
| Self-destructive biting H | ☐ Yes ☐ No ☐ Unknown | |
| Hyperactivity, restlessness H | ☐ Yes* ☐ No | |
| If yes*, please describe M |  | |
| Exophthalmos H | ☐ Yes ☐ No ☐ Unknown | |
| Tremors H | ☐ Yes ☐ No ☐ Unknown | |
| Muscle spasms H | ☐ Yes ☐ No ☐ Unknown | |
| Wet dog shakes ☐ Unknown | ☐ Yes ☐ No ☐ Unknown | |
| Stretching (Including writhing, extension) H | ☐ Yes ☐ No ☐ Unknown | |
| Retropulsion H | ☐ Yes ☐ No ☐ Unknown | |
| Arching H | ☐ Yes ☐ No | |
| Catatonia H | ☐ Yes ☐ No | |
| Startle responses H | ☐ Yes ☐ No | |
| Aggressiveness H | ☐ Yes ☐ No | |
| Corneal reflex (Including Eye twitch) H | ☐ Yes ☐ No | |
| Ptosis H | ☐ Yes ☐ No ☐ Unknown | |
| Mydriasis H | ☐ Yes ☐ No ☐ Unknown | |
| Pinnae reflex H | ☐ Yes ☐ No | |
| Visual tracking H | ☐ Yes ☐ No | |
| Vocalizations H | ☐ Yes ☐ No ☐ Unknown | |
| Grooming H | ☐ Yes ☐ No | |
| Anesthesia H | ☐ Yes ☐ No ☐ Unknown | |
| Hyperesthesia H | ☐ Yes ☐ No ☐ Unknown | |
| Loss of righting reflex H | ☐ Yes ☐ No | |
| Decreased grip strength H | ☐ Yes ☐ No | |
| Increased pain response (Vocalization or exaggerated response to tail pinch) H | ☐ Yes ☐ No | |
| Increased pain response H | ☐ Yes ☐ No ☐ Unknown | |
| Was Grimace score used? H | ☐ Yes ☐ No | |
| If Grimace score (0-5) used, attach scoring metric and/or description M |  | |
| Decreased pain response H | ☐ Yes ☐ No ☐ Unknown | |
| Other behaviors M | ☐ Yes* ☐ No ☐ Unknown | |
| If yes*, please describe M |  | |
| Total duration of observation (mins/hrs/days) H |  | |

**Instructions**: Please check boxes where applicable. If none of the predetermined options is appropriate use the default space to specify your answer.

This form is to be filled in for one individual animal.
